# Workplace Health Promotion and Mental Health: Three-Year Findings from Partnering Healthy@Work

**DOI:** 10.1371/journal.pone.0156791

**Published:** 2016-08-11

**Authors:** Lisa Jarman, Angela Martin, Alison Venn, Petr Otahal, Leigh Blizzard, Brook Teale, Kristy Sanderson

**Affiliations:** 1 Menzies Institute for Medical Research, Hobart, Australia; 2 Tasmanian School of Business and Economics, University of Tasmania, Hobart, Australia; 3 Department of Premier and Cabinet, Tasmanian State Government, Hobart, Australia; University of Exeter, UNITED KINGDOM

## Abstract

This study aimed to investigate the association between mental health and comprehensive workplace health promotion (WHP) delivered to an entire state public service workforce (~28,000 employees) over a three-year period. Government departments in a state public service were supported to design and deliver a comprehensive, multi-component health promotion program, Healthy@Work, which targeted modifiable health risks including unhealthy lifestyles and stress. Repeated cross-sectional surveys compared self-reported psychological distress (Kessler-10; K10) at commencement (N = 3406) and after 3 years (N = 3228). WHP availability and participation over time was assessed, and associations between the K10 and exposure to programs estimated. Analyses were repeated for a cohort subgroup (N = 580). Data were weighted for non-response. Participation in any mental health and lifestyle programs approximately doubled after 3 years. Both male and female employees with poorer mental health participated more often over time. Women’s psychological distress decreased over time but this change was only partially attributable to participation in WHP, and only to lifestyle interventions. Average psychological distress did not change over time for men. Unexpectedly, program components directly targeting mental health were not associated with distress for either men or women. Cohort results corroborated findings. Healthy@Work was successful in increasing participation across a range of program types, including for men and women with poorer mental health. A small positive association of participation in lifestyle programs with mental health was observed for women but not men. The lack of association of mental health programs may have reflected program quality, its universality of application or other contextual factors.

## Introduction

A recent meta-analysis based on 174 large-scale mental health surveys across 63 countries calculated that common mental disorders (CMD) (e.g. anxiety, depression, substance abuse) were experienced by 18% of adults within the past 12 months and 30% of adults over their lifetime [1]. Approximately two-thirds of people with a CMD are employed with significant repercussions for labour productivity and economic growth [2], health and welfare systems, community functioning and societal equity [3]. The cost of CMDs has been forecast at $16 trillion over 2012–2032 [2].

Reducing this burden of CMDs in the workforce requires a multi-component approach including both preventive and disease-management interventions [4,5]. Universal workplace interventions directly targeting mental health can be effective in reducing symptoms of depression [6,7]. There is also increasing recognition that occupational health programs targeting modifiable health risk factors such as physical activity and nutrition may also have benefits for mental health [8,9]. A meta-analysis has shown that employee mental health can benefit from health promotion interventions that either directly target mental factors or operate through indirect pathways focused on modifiable lifestyle risk factors (e.g. lifestyle choices, individual behaviours, changes to the work setting) [10].

Comprehensive workplace health promotion (WHP) simultaneously addresses a range of health-risk factors that may impact health, wellbeing and productivity. It considers mental and physical health (individual factors) as well as the need to make work structures supportive of health-promoting choices (organizational factors) [11]. As such this framework can include important mental health strategies such as access to mental health services, stigma elimination, and improved mental health literacy [12]. Evaluation research on interventions incorporating individual and organizational components is complex and challenging and as a result such studies are rare [13].

Benefits from WHP rely on well-designed, multi-component programmes that are sustained via an embedded health-promoting workplace culture [14]. To be effective in addressing chronic illness, WHP needs to include health screening, provide programmes addressing multiple risk factors (e.g. physical inactivity, smoking, stress and poor nutrition) [15] and be supported through work environment changes encouraging health promoting choices [16]. Good quality recruitment strategies into WHP programmes also play an important role so that there is broad employee participation. Programmes need to be available and accessible to participants [17], and attract people at risk of poor health rather than just selective participation from the ‘worried well’ [18].

A small number of studies have been published on comprehensive WHP but have: i) tended to focus on measuring lifestyle risk factors only [19]; ii) concentrated on a certain segment of an organization [20]; or iii) used proxy indicators of mental health such as job stress [21]. We were unable to identify any studies that evaluated the effects of comprehensive WHP in relation to mental health outcomes.

This article describes the Healthy@Work WHP initiative and assesses changes in population mental health over a 3-year period. Healthy@Work was based upon best-practice principles for comprehensive WHP [22], and implemented in a large and diverse public sector workforce located in regional Australia. Public sector (government) workers are of interest because research has shown mental health problems, including job stress are more prevalent in the public than private sector [23,24]. Our research questions were: i) which interventions were implemented in Healthy@Work that could have benefitted mental health; and ii) what was the association of these interventions with psychological distress over the 3-year evaluation period? We examined both the availability of (reach), and participation in (dose) WHP because positive mental health effects have been identified for health-promoting environments [15] as well as activity-based participation [25].

## Materials and Methods

### Study design

The study used a repeated, randomly-selected cross-sectional workforce survey design with a cohort subgroup occurring by chance. Survey measures have been described previously [26]. Ethics approval was provided by the Health and Research Ethics Committee (Tasmania) Network (ID: H0010501) and participants gave their informed consent in writing.

### Setting and description of Healthy@Work

This research was conducted in Tasmania, an Australian state with a population of around half a million people. In mid-2008, the Tasmanian Government made a 3-year commitment (2009–2012) to implement health and wellbeing programmes within its own public sector workforce, which was comprised of around 28,000 employees working around the state (urban, regional and remote) in a diverse range of organizations (e.g. health, education, police, forestry, electricity) and occupations. Over $2 million was committed to this ‘Healthy@Work’ project, which commenced in November 2008. A December 2008 initial audit of workplace health and wellbeing activities within this public sector workforce showed that 6 of its 15 government organizations (also called departments) had a program in place. The average number of initiatives per department increased from 13 to 48 after 3 years, with most increases reported in the final year [27, 28].

The goal of Healthy@Work was to support the development of health promotion programmes across its entire workforce that improved the health and wellbeing of all employees. It was intended to be a high quality program framework that was devolved to departments through a mandated directive from the elected head of government. Key values associated with Healthy@Work were equity of access, leadership commitment, sustainability, targeting of key priorities, organization-based strategies, framework flexibility and evaluation. Intended outcomes included:

1. improved health and wellbeing in relation to physical activity, nutrition, alcohol consumption, smoking and psychosocial factors (including mental health and stress),
2. increased employer and employee awareness of health and well-being issues,
3. improvement in workforce health and wellbeing policies and programmes within the Tasmanian Government. Programmes were to target the work environment as well as individuals,
4. employee-valued workplace health and wellbeing programmes, and
5. making healthy choices easy choices within the workforce.

### Participants

**Fig 1:**
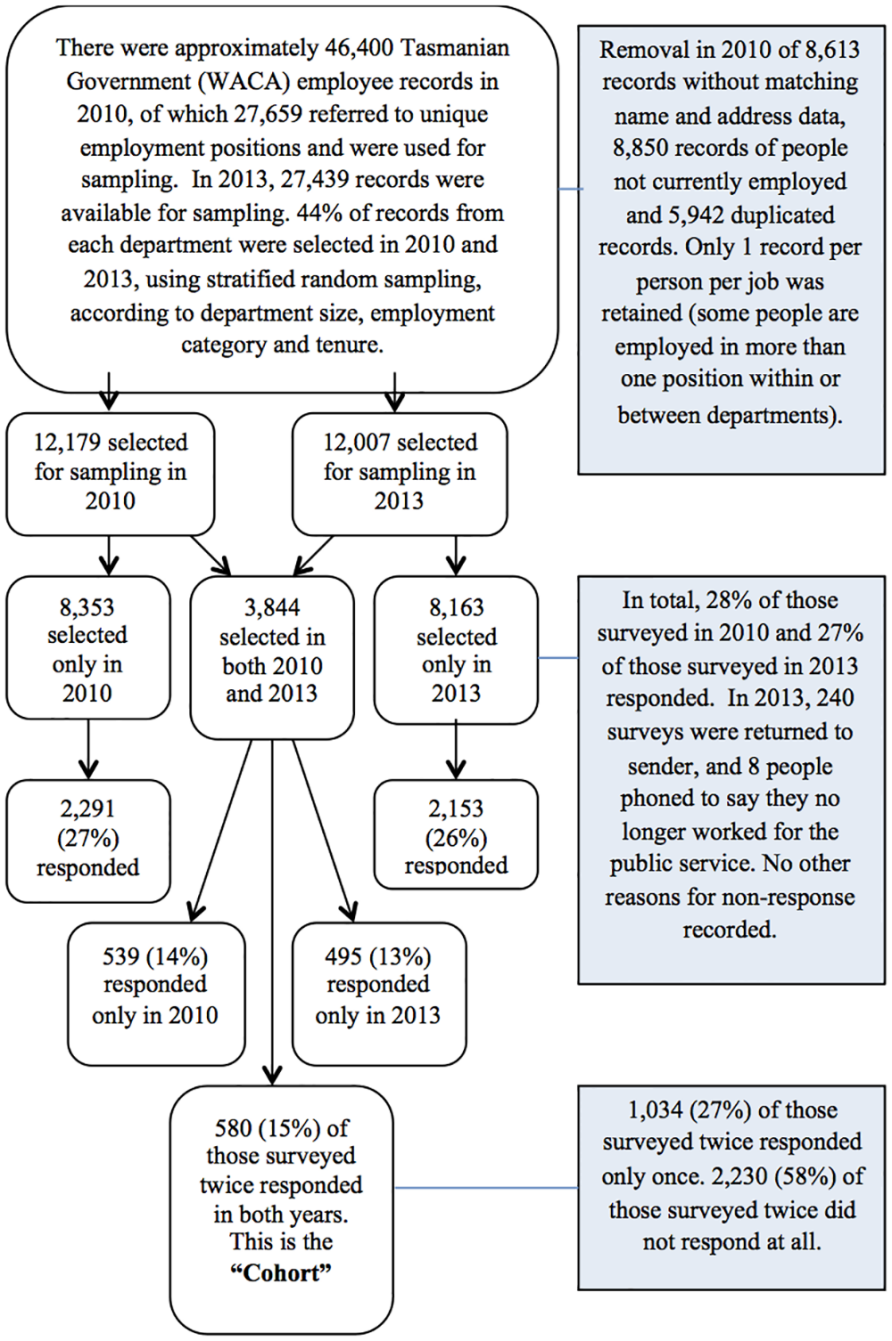
Flowchart showing sampling and responses to the Partnering Healthy@Work surveys as at November 2014.

For both of these self-report surveys we selected a 40% random population sample from the total pool of employees, stratified according to employment condition (permanent, fixed-term/ casual), employment category (full-time, part-time) across the departments. Survey responses were linked with administrative human resource data. By chance a portion of the population was re-surveyed and responded twice (men = 161; women = 423) and this group is referred to as the ‘cohort’. Fig 1 shows sampling processes and responses to these surveys.

### Variables

#### Outcome

The mental health outcome was psychological distress, which was measured using the Kessler-10 (K10), which ranges from 10 (lowest distress) to 50 (highest distress) [29]. Variants of the K10 have been used in employed cohorts [23,30] and the10-item has good precision detecting clinically diagnosable CMDs (e.g. anxiety and depression) in the high to very-high range of diagnosis [29].

#### Workplace Health Promotion initiatives (exposures)

In each survey (S1 Dataset. Partnering Healthy@Work dataset), participants were asked to report which Healthy@Work activities and supports were available in their workplace by giving a ‘yes’ or ‘no’ answer to a list of WHP initiatives that were listed separately in 2010 but then categorized into matched-item groups in 2013 as health assessments (e.g. health checks), mental health and well-being programs (e.g. stress management, employee assistance programs, relaxation, education), health education (e.g. seminars), physical activity programs (e.g. sessions, sports teams), injury prevention/rehabilitation, health policies (e.g. flexible work) and amenities (e.g. physical space for health-activities, healthy food options, access to stairs, health information bulletins). In 2010 the reporting time-frame was ‘the previous 12 months’ and in 2013 it was ‘previous 3 years’ to cover the period of the intervention (S1 Appendix. Question items used in the 2013 Partnering Healthy@Work survey to calculate workplace health promotion exposures for availability and participation). Where a respondent gave a ‘yes’ answer, they were also asked to write the number of times they had participated in activities.

We counted the number of positive responses each person provided for the availability question to give an indication of total availability of Healthy@Work and calculated times participated to represent participation. Total availability was classified into one of 3 types of WHP intervention: 1) *mental health*—availability of activities directly targeting individual mental health and well-being (e.g. mental health education, stress management); 2) *lifestyle*—individually targeted activities targeting known risk factors associated with poor mental health (i.e. health education, health assessments, physical activity, injury support); 3) *organizational*—workplace initiatives indirectly targeting mental health (i.e. changes to amenities, health-promoting policies). System interventions targeting work organization and psychosocial factors were not recorded as they were not included in Healthy@Work. Questions about organizational initiatives targeted perceptions of factors contributing to a health promoting setting rather than the participation itself, so participation was classified into mental health and lifestyle categories only, which were then added together to form a mean total participation score.

### Statistical analysis

Analyses were stratified by sex due to known differences in the way that men and women self-report mental health [31], which were evident in the 2010 survey data [26]. Repeated cross-sectional analysis (described in more detail below) was treated as a two-stage process involving i) assessing whether WHP availability and participation changed over time, and ii) assessing whether mean K10 scores were different over time and then estimating associations between the K10 and exposure to Healthy@Work programs at 2010 and 2013. Survey responses were anticipated to be more similar within than between government departments, and for those who were repeat respondents. Accordingly we used mixed-effects linear modelling with random intercepts for department and participants to allow for related responses. In stage 1, we used Poisson regression with random effects to assess whether mean reported availability of WHP programs or participation in those programs had changed over time. These regressions derived ratios of the means for these exposures to WHP in 2013, relative to 2010. Model diagnostics from linear mixed effects models showed that residuals were skewed and an inverse transformation was applied to the K10 values. We then back-transformed the K10 to present mean estimates on the original scale of measurement. Further we applied propensity weighting as described by Little and Rubin [32] to deal with potential non-response bias; the propensity model included age, sex, government department, employment category, employment condition, and tenure using the human resources administrative database as the reference population.

In stage 2, models were constructed with the outcome K10 and a dummy variable for *‘survey year’* in the fixed effect section of each model along with covariates for confounders. This process allowed us to determine whether psychological distress scores differed by survey year. We then constructed mixed models by adding the exposure variables and covariates. Covariates were identified via regression modelling techniques described by Hosmer, Lemeshow and Sturdivant [33] and were defined as those variables that were associated with the outcome and which also produced more than 10% change in an estimated coefficient of the model. Tested covariates included age, marital status (married/living with partner, not married), annual salary, employment category, employment condition, tenure or hours worked. We tested for interaction between survey year and exposure variables in each model to assess whether the effect of exposure differed between surveys. Models showing relationships between the exposure and outcome were corroborated by replicating the analysis with the cohort group. All analyses were conducted using STATA 12.1 (StataCorp LP, Texas, USA).

## Results

### Participants

The overall response proportions for the surveys were 28% (n = 3408) in 2010 and 27% in 2013 (n = 3228). The proportions of men and women were also similar across both time points (women: 2010 = 67%; 2013 = 68%). Table 1 shows that employees returning surveys had similar characteristics across both time-points.

**Table 1:** Respondent characteristics for the 2010 and 2013 Healthy@Work surveys.

	Men					Women				
	2010		2013			2010		2013		
**Continuous Variables**	**Mean**	**SD^1^**	**Mean**	**SD**	***p*^3^**	**Mean**	**SD**	**Mean**	**SD**	***p***
*Age [years*, *mean] (SE)*	47.1	10.1	47.6	10.4	0.677	45.8	10.4	46.8	10.3	0.011
*Tenure (SE)*	14.1	11.8	14.9	11.7	0.156	12.7	10.2	13.0	10.3	0.238
*Hours worked (SE)*	40.4	12.9	40.1	13.4	0.990	36.8	15.7	36.0	15.6	0.085
**Categorical Variables**	**%**	**N**^2^	**%**	**N**		**%**	**N**	**%**	**N**	
*Marital Status*										
Married/ Partner	91	774	94	763	ref	85	1767	85	1711	ref
Not married	9	79	6	52	0.022	15	313	15	301	0.873
*Education*										
Post school	61	495	66	514	ref	68	1308	66	1239	ref
Middle school	3	23	2	12	0.190	2	40	2	36	0.651
Upper school	36	291	32	248	0.115	30	567	32	606	0.143
*Employment Category*										
Permanent	88	848	86	785	ref	92	2256	88	2034	ref
Fixed-term/ Casual	12	116	14	131	0.893	8	188	12	276	<0.001
*Employment Condition*										
Full Time	84	814	84	772	ref	51	1243	48	1105	ref
Part Time	16	150	16	144	0.474	49	1201	52	1206	<0.001
**Total Respondents**		964		917			2444		2311	

^1^ Standard Deviation

^2^ Number of respondents

^3^ p-value has been weighted for non-response

### Availability of workplace health promotion over time

The mean reported availability of these programs was 14% higher in 2013 than in 2010 for both men and women (Table 2), whereas the mean reported availability of specific mental health programs in 2013 was 10% less for men (*p*<0.024) and stable for women (*p* = 0.604). Mean reported availability of lifestyle programs was more than 50% greater for both men and women in 2013. The mean reported availability of organizational interventions was slightly greater for men *(p* = 0.022) and women (*p*<0.001) in 2013 than 2010.

**Table 2:** Ratios of mean reported availability and participation in Healthy@Work initiatives in 2013 relative to 2010.

Healthy@Work Exposure	Men						Women					
	Mean		MeanRatio^1^	95% CI		*p*	Mean		Mean Ratio	95% CI		*p*
*WHP Availability*	*2010*	*2013*					*2010*	*2013*				
Total	0.41	0.46	1.14	1.10	1.19	<0.001	0.38	0.42	1.14	1.11	1.17	<0.001
Mental Health^2^	0.47	0.46	0.90	0.82	0.99	0.024	0.45	0.46	1.02	0.94	1.11	0.604
Lifestyle^3^	0.29	0.43	1.55	1.43	1.67	<0.001	0.26	0.37	1.52	1.45	1.60	<0.001
Organization^4^	0.41	0.43	1.05	1.01	1.10	0.022	0.38	0.40	1.06	1.03	1.10	<0.001
*WHP Participation*												
Total	1.97	4.85	1.93	1.71	2.17	<0.001	1.66	3.77	2.16	2.00	2.34	<0.001
Mental Health	0.42	0.59	1.61	1.30	2.00	<0.001	0.29	0.51	1.89	1.61	2.20	<0.001
Lifestyle	1.55	4.27	2.16	1.89	2.47	<0.001	1.37	3.25	2.26	2.07	2.46	<0.001

^1^ Data were propensity weighted for non-response. This process results in estimated rather than actual means. Thus ratios of the estimated means for reports of exposure to availability of, and participation in Healthy@Work initiatives in 2013, relative to 2010 are presented.

^2^ Mental health interventions refer to initiatives directly targeting individual mental health, including stress management programs, employee assistance programs, relaxation etc.

^3^ Lifestyle interventions refer to interventions targeting individual risk factors known to be associated with poor mental health such as inactivity, nutrition, and high alcohol consumption.

^4^ Organizational strategies are also a form of indirect mental health intervention and refer to a supportive physical environment (e.g. activity space, healthy food options, access to stairs), health promoting policies and individual-organizational initiatives.

### Mean participation over time

Overall, men reported participating in 93% more programs in 2013 than in 2010 while women reported participating in 116% more programs (Table 2). The increase in participation was slightly greater in lifestyle compared with mental health programs.

### Univariable correlates of psychological distress

Both sexes had the following covariates univariably associated with psychological distress: age, marital status, annual salary, employment category and employment condition (p < 0.25). Hours worked was an additional covariate for women (Table 3).

**Table 3:** Univariable associations between psychological distress (Kessler-10) and respondent characteristics stratified by sex and survey year.

	Men						Women					
	2010			2013			2010			2013		
Kessler-10	ß	95% CI		ß	95% CI		ß	95% CI		ß	95% CI	
*Age (continuous)*	-0.045	-0.066	-0.023	-0.028	-0.049	-0.008	-0.060	-0.074	-0.047	-0.040	-0.050	-0.020
*Marital status*												
Married	ref			ref			ref			ref		
Not married	0.141	-0.719	1.001	1.039	-0.233	2.311	0.110	-0.366	0.585	0.376	-0.131	0.883
*Education*												
Post school	ref			ref			ref			ref		
Middle school	-0.761	-2.151	0.628	0.325	-2.458	3.107	0.487	-1.134	2.108	-0.116	-1.199	0.967
Upper school	-0.029	-0.533	0.474	0.084	-0.430	0.598	-0.007	-0.384	0.370	0.595	0.197	0.994
*Employment category*												
Permanent	ref			ref			ref			ref		
Fixed term/casual	0.387	-0.393	1.167	-0.855	-1.418	-0.292	0.172	-0.373	0.717	0.479	-0.054	1.013
*Employment condition*												
Full-time	ref			ref			ref			ref		
Part-time	0.093	-0.572	0.758	-0.496	-1.077	0.084	-0.331	-0.627	-0.035	-0.299	-0.620	0.021
*Tenure*	-0.028	-0.046	-0.010	-0.018	-0.037	0.001	-0.041	-0.056	-0.027	-0.036	-0.051	-0.021
*Hours worked (continuous)*	0.001	-0.017	0.020	0.009	-0.008	0.025	0.008	-0.002	0.018	0.017	0.006	0.027

### Repeated cross-sectional modelling

#### Changes in psychological distress over time

Table 4 shows that women’s K10 scores were lower on average in 2013 than in 2010 (p = 0.007) and for the subgroup who were participants in Healthy@Work programs (p = 0.009). No changes in K10 scores over time were observed for men.

**Table 4:** Linear mixed models (adjusted) regressing psychological distress (Kessler-10) on survey year and on different types of reported exposure to Healthy@Work.

	Men							Women						
	2010			2013				2010			2013			
**Kessler-10**	**β**^1^	**95%CI**		**β**	**95%CI**		***p***^3^	**β**	**95%CI**		**β**	**95%CI**		***p***
*All respondents (mean)*^2^^a^	12.76	12.33	13.18	12.87	12.55	13.20	0.282	14.27	14.08	14.47	14.08	13.91	14.26	0.007
*Participants*^b^	15.00	14.66	15.34	14.94	14.64	15.24	0.784	14.37	14.10	14.64	14.10	13.91	14.29	0.009
**Healthy@Work Exposure**	**β**^4^	**95%CI**		**β**	**95%CI**		***p***^4^	**β**	**95%CI**		**β**	**95%CI**		***p***
*WHP Availability*	(~n = 947)			(~n = 906)				(~n = 2396)			(~n = 2283)			
Total ^a^	-0.006	-0.157	0.144	-0.007	-0.159	0.146	0.997	-0.004	-0.114	0.105	-0.034	-0.127	0.060	0.228
Mental Health^c^	-0.087	-0.843	0.669	-0.088	-0.856	0.679	0.931	-0.026	-1.105	1.054	-0.006	-1.071	1.058	0.064
Lifestyle^a^	-0.137	-0.397	0.122	-0.140	-0.407	0.128	0.786	-0.015	-0.142	0.113	-0.004	-0.128	0.120	0.877
Organization^a^	0.031	-0.195	0.256	0.031	-0.195	0.257	0.828	-0.036	-0.132	0.059	-0.025	-0.116	0.066	0.372
*WHP Participation*	(~n = 735)			(~n = 759)				(~n = 1804)			(~n = 1881)			
Total^b^	-0.018	-0.057	0.022	-0.018	-0.056	0.021	0.378	0.033	0.010	0.057	0.040	0.016	0.063	0.630
Mental Health^d^	0.007	-0.058	0.072	0.007	-0.058	0.072	0.863	0.024	-0.069	0.118	0.031	-0.059	0.121	0.225
Lifestyle	-0.026	-0.089	0.038	-0.025	-0.087	0.037	0.497	0.035	0.005	0.064	0.041	0.012	0.070	0.333

^1^ Kessler 10 coefficient estimated at mean after back-transformation and controlling for confounders.

^2^ Linear mixed model regresses psychological distress on survey year.

^3^ The probability value compares population means of psychological distress scores over time and is derived from the linear mixed models.

^4^ Beta values represent the results from linear mixed models including reported exposures to WHP initiatives but with no interaction term (i.e. additive models).

^a^ Adjusted: Men–age (estimated mean) and employment condition; Women–age

^b^ Adjusted: Men–age and marital status; Women–age

^c^ Adjusted: Men–age and employment condition; Women–age and marital status

^d^ Adjusted: Men–age; Women–age and tenure

#### Associations between availability and psychological distress

Table 4 highlights that there was no relationship between reported availability of workplace health promotion and psychological distress by sex in either 2010 or 2013. When the components of availability were considered, no associations were found by sex for mental health or lifestyle programs, or organizational interventions.

#### Associations between participation and psychological distress

Table 4 shows a modest positive association over time (β = 0.038 [95% CI: 0.011 to 0.064]) between the total number of times women participated and psychological distress after adjusting for age. This relationship appeared largely due to participation in lifestyle programs (β = 0.037 [95% CI: 0.006 to 0.068]) because no clear association was identified for women for mental health programs (β = 0.088 [95% CI: -0.037 to 0.210]). However we note that the confidence intervals presented for mental health programs were close to zero. For men, no association was identified between participation and psychological distress (p = 0.378). No statistical interactions were present between Healthy@Work participation and survey year in any models.

### Cohort analyses

When we replicated the models with the cohort the results across each analysis were consistent with the effects observed for both reported availability and reported participation models in the larger respondent population (Table 5).

**Table 5:** Linear mixed models (adjusted) regressing psychological distress (Kessler-10) on survey year and on different types of reported exposure to Healthy@Work for the cohort of repeat survey responders (n = 580).

	Men							Women						
	2010			2013				2010			2013			
**Kessler-10**	**β**^1^	**95% CI**		**β**	**95% CI**		***p***^3^	**β**	**95% CI**		**β**	**95% CI**		***p***
*All respondents (mean)*^2^^a^	12.32	11.77	12.87	12.46	12.03	12.89	0.197	14.12	13.78	14.46	13.91	13.53	14.29	0.036
*Participants only*^b^	14.34	13.66	15.02	14.31	13.62	14.99	0.848	14.19	13.79	14.59	13.90	13.56	14.23	0.002
**Healthy@Work Exposure**	**β**^4^	**95% CI**		**β**	**95% CI**		***p***^4^	**β**	**95% CI**		**β**	**95% CI**		***p***
*WHP Availability*														
Total ^a^	<-0.001	-0.141	0.140	<-0.001	-0.144	0.143	0.995	-0.027	-0.139	0.086	-0.019	-0.124	0.086	0.250
Mental Health^c^	-0.094	-0.834	0.647	-0.096	-0.850	0.659	0.920	-0.019	-1.250	1.211	0.003	-1.213	1.220	0.072
Lifestyle^a^	-0.132	-0.392	0.128	-0.135	-0.403	0.134	0.775	-0.005	-0.148	0.138	0.002	-0.130	0.134	0.843
Organization^a^	0.039	-0.176	0.253	0.039	-0.181	0.260	0.834	-0.027	-0.131	0.078	-0.017	-0.117	0.083	0.415
*WHP Participation*														
Total^b^	-0.020	-0.072	0.032	-0.020	-0.071	0.031	0.229	0.034	0.008	0.060	0.038	0.018	0.059	0.599
Mental Health^d^	0.006	-0.057	0.069	0.006	-0.057	0.069	0.860	0.028	-0.068	0.123	0.032	-0.064	0.128	0.246
Lifestyle	-0.035	-0.137	0.067	-0.034	-0.135	0.066	0.303	0.035	0.002	0.068	0.039	0.013	0.066	0.318

^1^ Kessler-10 coefficient estimated at mean after back-transformation and controlling for confounders.

^2^ Linear mixed model regresses psychological distress on survey year.

^3^ The probability value compares population means of psychological distress scores over time and is derived from the linear mixed models.

^4^ Beta values represent the results from linear mixed models including reported exposures to WHP initiatives but with no interaction term (i.e. additive models).

^a^ Adjusted: Men–age (estimated mean) and employment condition; Women–age

^b^ Adjusted: Men–age and marital status; Women–age

^c^ Adjusted: Men–age and employment condition; Women–age and marital status

^d^ Adjusted: Men–age; Women–age and tenure

## Discussion

While the total reported availability of WHP initiatives increased over time, employees reported no increases in availability of programs directly targeting mental health and modest increases in organizational strategies such as health-promoting amenities or policies. However reported availability of lifestyle-related programs increased by more than 50% for men and women. Participation in 2013 was approximately double that of 2010 for both lifestyle and mental health programs in both men and women. Other research from this project found that workers who had variable work schedules, those who smoked, or who had cardio-metabolic problems were less likely to participate despite activities being available. Participation was more common among administrative employees and workers who undertook leisure-time physical activity [34]. We surmise that over time government departments were indirectly promoting mental health through organizational strategies (e.g. physical environments, policies), which may have enhanced participation. However, consistently higher volumes of mental health programs across the working population were possibly unavailable until the last year of Healthy@Work, and even then many employees may not have reported program availability due to factors such as job type or communication strategy adequacy.

Our results established that psychological distress was less common in 2013 than in 2010 for women. At both time points women with higher K10 scores (higher levels of distress) also tended to participate more in workplace health promotion programs. Contrary to the ‘inequality paradox’, which suggests that workers who have better mental health will participate more [35], this finding indicated that the intervention attracted participation from women with poorer mental health. This association appeared due to participation in lifestyle-related (indirect) forms of mental health promotion, such as physical activity and health education. However the small association between women’s participation and psychological distress did not fully explain the reduction in K10 scores. Our results were corroborated through sub-analyses using the cohort of repeat responders, which provided further support for these findings. Recent data from the general working population in the same region showed psychological distress scores were stable for men and women over the same period [36], suggesting that workplace rather than societal factors may have contributed to our results.

It is possible that improvements in mental health for women were associated with reductions in work stressors (organizational change, psychosocial risks) that occurred at the same time Healthy@Work was implemented. These types of organizational interventions were not part of Healthy@Work and so were not quantitatively recorded during our survey processes. It is also possible that women had more opportunities for participation, or were more motivated to participate after 3 years. These are areas needing further investigation. As was noted in the introduction, small effects on mental health from WHP were expected as greater impact will come from a multi-level approach [4]. But any modest improvement can be important given there are many other positive reasons to implement comprehensive WHP [14].

Male participants had higher mean K10 scores than non-participants in both survey years (Table 4). However the mental health programs perceived to be available by men decreased over time. We had expected the most obvious pathway for association would be via exposure to programs directly targeting mental health. A number of factors that could have influenced the mental health program results including marketing, content and quality, pre-existing participant mental health [37], or mental health literacy [38]. Future research in naturalistic environments would benefit from closer examination of these factors. However systematic differences have also been found for men and women in the way they perceive work and report on work [39]. For men, who were higher wage-earners and more likely to be in full-time or management positions in this organization, perceived or real exposure to threats of job-loss and work intensification [40] may have contributed to results. Selected or indicated interventions [41], employing effective mental health programs may have been more appropriate for men in this environment.

Healthy@Work was very successful in attracting participation from employees. Data from other studies of universal programs shows that quality mental health programs delivered through WHP can improve employee mental health [7]. Further, as mental illness is frequently a covert disorder, the observed increases in participation in mental health programs are a good sign and may reflect decreased stigma [42,43]. Clearly further inquiry is needed to determine why these direct programs in this study did not translate to a change in population mental health. For studies of whole working populations, it may be that an integrated approach to mental health surveillance that includes health protection, promotion and job-specific interventions is needed to better understand the underlying dynamics between these intervention areas.

### Limitations

The repeated cross-sectional and cohort subgroup analyses produced consistent findings, but neither approach allows causal inference. However, the choice of a repeated-cross sectional design does not infer, *a priori*, that the findings presented here were biased. Given our attention to sample size, random stratified sampling, weighting procedures and choice of analysis we can reasonably expect that these results may be generalizable to similar populations of public sector workers. Our response rates were arguably typical for organizational surveys [23] but these proportions only become an issue if missing data are non-random. We used propensity weighting to minimize bias due to non-response but acknowledge that some people with poor mental health may have chosen not to respond to the surveys [44]. Furthermore, the large random survey samples generated a high number of responses at each time point and as such we can be reasonably confident of their generalizability to the working population under study. Some factors that could have influenced employee mental health were not measured, including the changing nature of work and organizational context. Factors such as the presence of remote worksites or high workforce proportions of part-time or shift-work, may have had different types of effects on how WHP was experienced [45].

We note that our self-reported measures of availability and participation were susceptible to recall bias [46] and we have discussed the challenges inherent to Healthy@Work’s measurement elsewhere [28]. In that publication we note how the design was able to deal with the heterogeneity of comprehensive WHP, and enabled us to capture the full period of WHP. Ultimately we cannot say whether the changes in wording of the response period for our exposures affected the results or if the time period was adequate to capture exposure effects. The self-reported increases in WHP availability appeared to reflect increases in departmental data obtained from the employers’ audit processes (S1 Appendix. Question items used in the 2013 Partnering Healthy@Work survey to calculate workplace health promotion exposures for availability and participation) and this is an area needing further investigation. Measurement of WHP programs and organizational interventions at a work unit level [47] may also have provided further clues about how factors such as manager support and operational priorities influenced individual reports of WHP availability and participation.

### Conclusions

Healthy@Work was successful in attracting participation from men with higher average psychological distress and increasing participation among women with poorer mental health scores. While these contributions were important, they did not translate to a change in men’s mental health and only made a partial contribution to the observed reduction in women’s psychological distress over time. Nevertheless, scope remains for comprehensive WHP to prove its worth as a universal intervention for mental health because direct interventions have evidence of success [47], and because they provide a pathway that raises the profile of mental health, thereby reducing its stigma [48]. When conducting naturalistic studies on mental health in work environments, a more integrated approach to employee health surveillance may be needed, which encompasses worker health promotion, protection and job-specific interventions.

## Supporting Information

S1 Appendix Question items used in the 2013 Partnering Healthy@Work survey to calculate workplace health promotion exposures for availability and participation. (ZIP)

S1 Dataset Partnering Healthy@Work dataset. (DTA)
