# Supplementary figures and images for: Workplace Health Promotion and Mental Health: Three-Year Findings from Partnering Healthy@Work
Source: PLoS One. 2016 Aug 11;11(8):e0156791. doi: 10.1371/journal.pone.0156791 (PMC4981343; doi:10.1371/journal.pone.0156791)

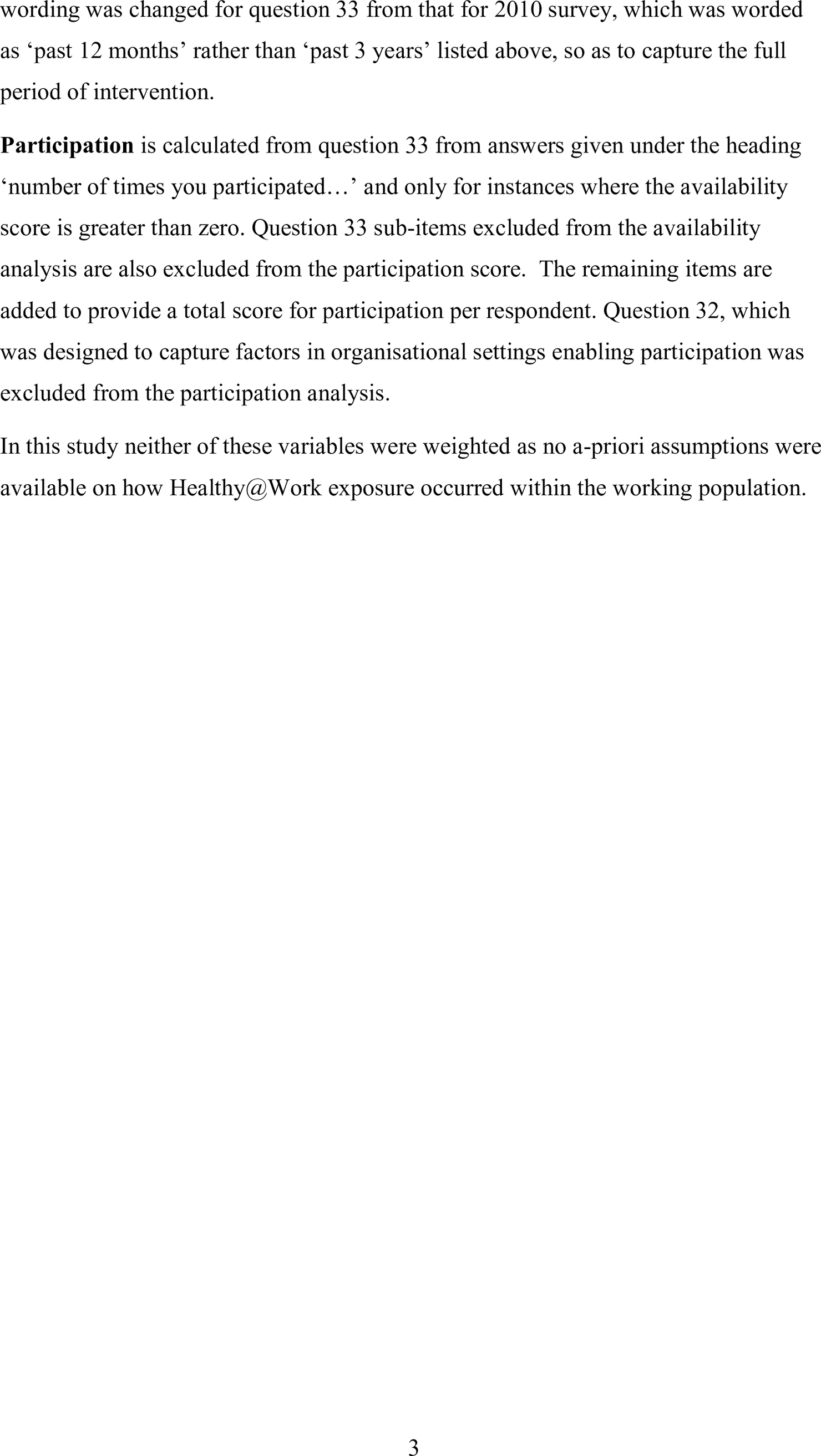

Supplement: S1 Appendix — (ZIP) [file pone.0156791.s001.zip › S1_Appendix-3.tif]

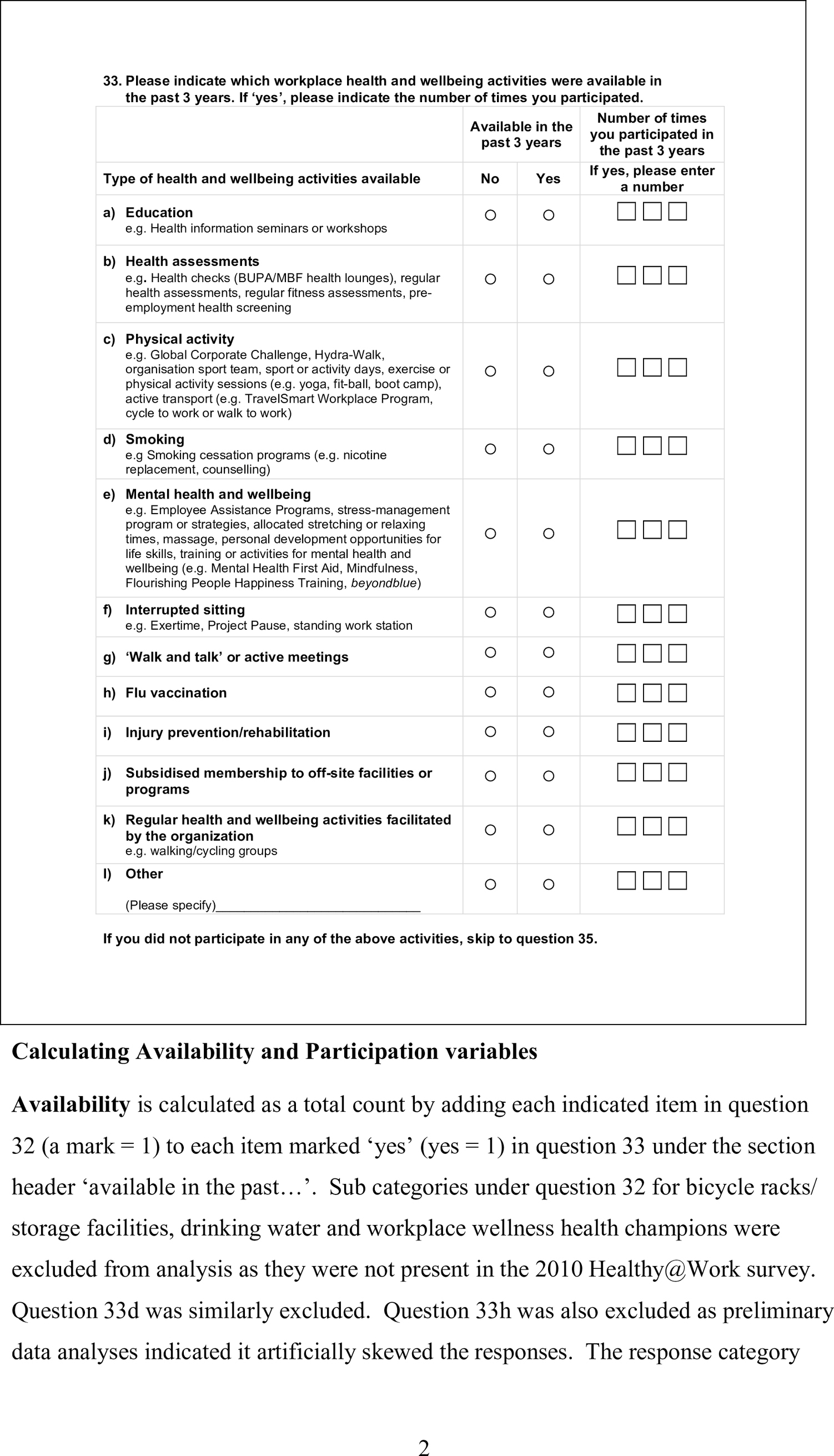

Supplement: S1 Appendix — (ZIP) [file pone.0156791.s001.zip › S1_Appendix-2.tif]

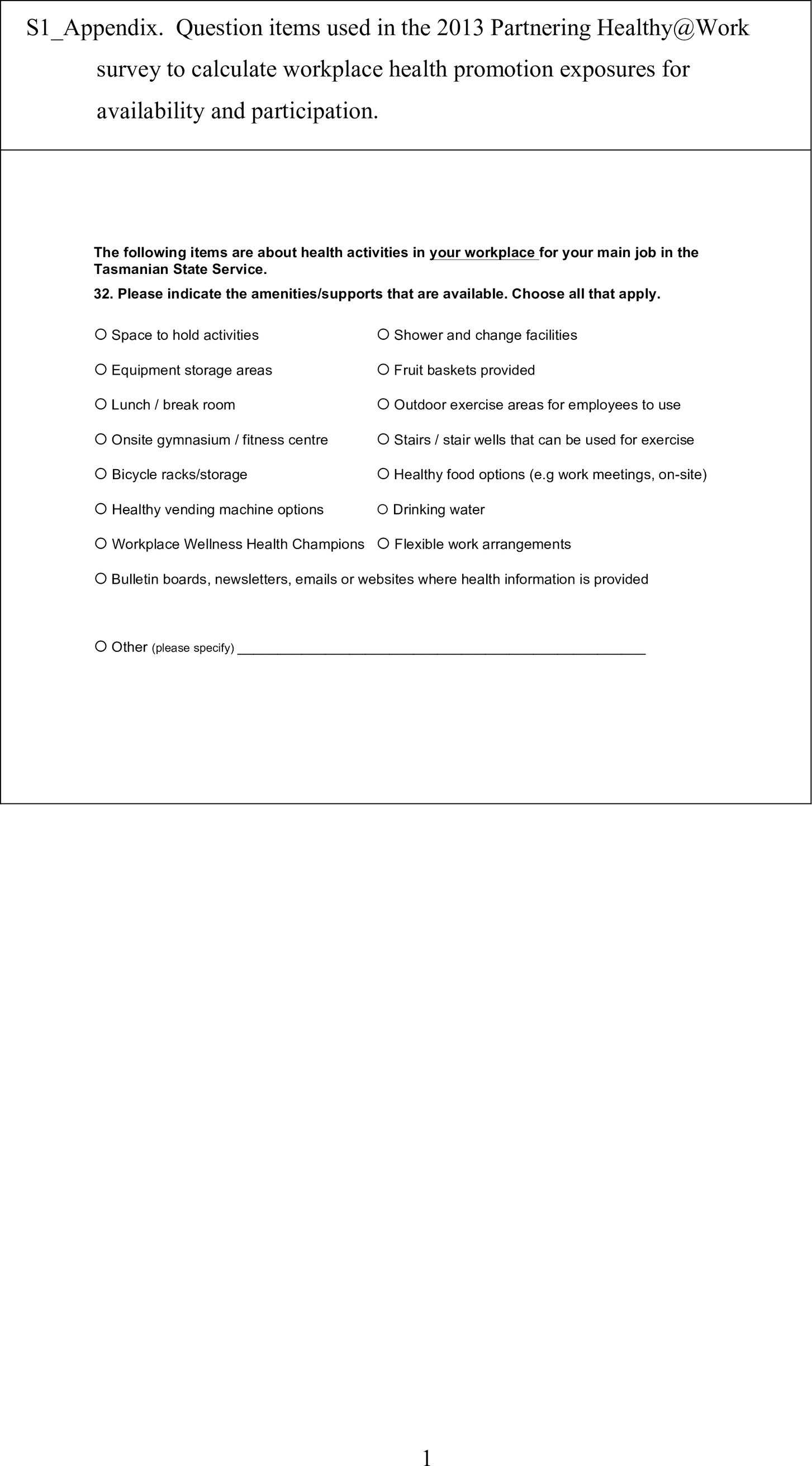

Supplement: S1 Appendix — (ZIP) [file pone.0156791.s001.zip › S1_Appendix-1.tif]
